# Epidemiological characteristics and mortality risk factors among COVID-19 patients in Ardabil, Northwest of Iran

**DOI:** 10.1186/s12873-021-00463-x

**Published:** 2021-06-02

**Authors:** Davoud Adham, Shahram Habibzadeh, Hassan Ghobadi, Shabnam Asghari Jajin, Abbas Abbasi-Ghahramanloo, Eslam Moradi-Asl

**Affiliations:** 1 Department of Public Health, School of Health, Ardabil University of Medical Sciences, Ardabil, Iran; 2 Department of Infection Diseases, Faculty of Medicine, Ardabil University of Medical Sciences, Ardabil, Iran; 3 Department of Internal Medicine, Pulmonary Division, Faculty of Medicine, Ardabil University of Medical Sciences, Ardabil, Iran

**Keywords:** COVID-19, Extra-pulmonary, Mortality, Iran

## Abstract

**Background:**

Coronavirus disease highly contagious, is prevalent in all age and sex groups infecting the respiratory system. The present study seeks to investigate the epidemiology and effective factors in mortality of patients with COVID-19 in Ardabil province, northwestern Iran.

**Methods:**

In a retrospective study, the hospitalized patients with laboratory-diagnosed COVID-19 between February to August 2020 were enrolled. The data registration portal was designated according to Iranian Ministry of Health and Medical Education guidelines. In this portal, demographic information, clinical presentation, laboratory and imaging data were registered for patients in all hospitals in the same format. The Hosmer-Lemeshow strategy was used for variable selection in a multiple model.

**Results:**

Of the patients involved 2812(50.3%) were male and 150 (2.7%) had contact with a confirmed case of COVID-19 in the last 14 days. Pre-existing comorbidity was reported in 1310 (23.4%) patients. Of all patients, 477(8.5%) died due to COVID-19. the result of the multiple logistic regression model indicated that after adjusting for other factors, higher age (OR = 3.11), fever or chills (OR = 1.61), shortness of breath (OR = 1.82), fatigue (OR = 0.71), headache (OR = 0.64), runny nose (OR = 1.54), Skeletal muscle pain (OR = 1.53), hospitalization (OR = 5.66), and hospitalization in ICU (OR = 5.12) were associated with death.

**Conclusions:**

Hospitalization had the strongest effect on mortality followed by hospitalization in ICU, and higher age. This study showed that having some extra-pulmonary symptoms in contrast with pulmonary symptoms can predict as good prognostic factors.

## Background

A Coronavirus is a group of single-stranded, enveloped RNA viruses with a diameter of 120–180 nm and is divided into four groups: Alpha, Beta, Gamma, and Delta. COVID-19 is a member of the Beta-coronavirus family [1, 2]. This type of virus is highly contagious and infects the respiratory system. Direct contact and respiratory droplets are the most common way of transmitting the virus in the community but other ways have also been suggested [3].

COVID-19 is associated with the demographic situation of the community. The disease is prevalent in all age and sex groups, but the highest mortality is in older men with an average age of 75 years who have a history of diseases such as cardiovascular diseases, diabetes, high blood pressure, chronic respiratory diseases, cancer,or previous surgery [4, 5]. According to the World Health Organization’s (WHO) weekly report, as of October 4, 2020, there have been 35,347,404 confirmed cases of the disease of which 1,039,406 were mortality rate the highest number it reported at 208,433 and 103,569 in the United States and India [6]. According to various studies, 18 to 33% of patients admitted to the hospital need mechanical ventilation, and up to 20% of patients are admitted to the ICU [7–10].

The most common symptoms of the disease include fever, cough, shortness of breath, fatigue, and muscle pain that are observed in most patients, and other symptoms such as diarrhea, headache and nausea have also been reported with a low percentage [11, 12]. Given that no vaccine has been successfully developed to prevent COVID-19 to date, public health measures to control the infection are necessary to limit the global spread of the virus to reduce the incidence of COVID-19. It is essential to limit travel, human-to-human transmission to reduce secondary infections in close contact with health care personnel and to prevent further spread of the disease. Many efforts have been made to slow the progression of the disease to provide or buy time for better public health care systems, a better description of COVID − 19 to guide public health advice, and timely development of diagnosis, treatment, and vaccines [13–15]. With the discovery of the vaccine and the start of vaccination, it is hoped that the incidence and mortality rate of this disease will be reduced. However, it is predictable that people have some uncertainty around the details of COVID-19 vaccination. Because of some domestic reasons and lack of vaccines, adherence to protocols is still emphasized in Iran [16]. In some countries such as Iran, the death toll is still partially intermediate. This study aims to investigate the epidemiology and mortality risk factors among COVID-19 patients in Ardabil province, northwestern Iran so that by identifying these factors we can provide basic proceedings for its control and prevention.

## Materials and methods

This retrospective cohort study was performed on 5587 patients in Ardabil, northwest of Iran. All patients who were admitted to the hospitals of Ardabil province from February to August 2020 were recruited for the study. During this pandemic, ten hospitals were allocated for COVID-19 in Ardabil province. All needed data were registered in the COVID-19 case registration portal at Ardabil University of Medical Sciences. The data were obtained retrospectively from this portal. The data registration portal was designated according to Iranian Ministry of Health and Medical Education guidelines. In this portal, demographic information, clinical presentation, laboratory and imaging data were registered for patients in all hospitals in the same format. The first part of this data assessed demographic information such as name, age, sex, residency place, etc. The second part evaluated information about the various clinical presentation of COVID-19 some related factors including experienced symptoms of the disease, history of contact with a confirmed case of COVID-19 in the last 14 days, the status of hospitalization, and hospitalization in ICU. The registered symptoms of COVID-19 were pulmonary symptoms such as fever or chills, cough, and shortness of breath, also, extra-pulmonary symptoms such as fatigue, sore throat, headache, runny nose, diarrhea, and skeletal muscle pain. Additionally, there was some information about the outcome of the COVID-19 and the type of treatment.

Having some preexisting chronic diseases was considered as a comorbidity in this study. These diseases were a chronic respiratory disease, cardiovascular disease, diabetes, liver disease, kidney disease, and malignancy. Atypical findings for COVID-19 on chest x-ray especially on chest CT such as an old scar, lymphadenopathy, and linear opacity does not interpret as COVID-19 imaging findings. Other abnormal findings were considered as possible signs of COVID-19.

The patients who were diagnosed with the COVID-19 according to the Iranian Ministry of Health and Medical Education guidelines have been registered in the mentioned portal. All patients with COVID-19 were eligible to enter this study and there were no exclusion criteria. The outcome was defined as death (non-survivor) or discharge (survivor). The criteria for discharge were the improvement of the general status and respiratory symptoms, absence of fever for at least 3 days, chest CT scan improvement, or at least one-time throat-swab or nasal-swab samples negative for SARS-CoV RNA assessment.

The data from all hospitals were recorded in the portal in the same format and the central office in Ardabil University of Medical Sciences had access to all recorded data anytime.

To perform simple statistical analysis, Chi-square and Fisher’s exact test was used to evaluate the relationship among hospitalization, hospitalization in the ICU, and outcome of disease with the related factors. Univariate and multiple logistic regression models were run to examine the correlation among different variables and death due to COVID-19. The Hosmer-Lemeshow guidelines were used for variable selection in a multiple model [17]. *P*-value < 0.05 was considered statistically significant.

## Results

The number of all registered patients was 5586. This study indicated that the mean age of the patients was 52.25 ± 20.21 years. Of all patients, 2812(50.3%) were male and 150 (2.7%) had contact with a confirmed case of COVID-19 in the last 14 days. Pre-existing morbidity was reported in 1310 (23.4%) patients. Of all patients, 477(8.5%) died due to COVID-19. Among all patients, 4188 (75.0%) were hospitalized and 124 (2.22%) were hospitalized in the ICU.

Table 1 shows the frequency of demographic characteristics and related factors to hospitalization, hospitalization in ICU, and the outcome of the disease. As seen in the table, hospitalization and the outcome of the disease had a significant correlation with all studied variables (*p* < 0.05). Hospitalization in ICU was significantly was correlated with age, comorbidity, the outcome of the disease, and abnormal findings in chest radiography. However, hospitalization in ICU didn’t have a significant correlation with sex and previous contact with a confirmed case of COVID-19 (*p* > 0.05).

**Table 1:** Basic characteristics of patients according to hospitalization and disease status in Ardabil province, Northwest of Iran,2020

Items	Hospitalization		Hospitalization in ICU		Outcome		
	Yes (%)	*P* value	Yes (%)	P value	Survive	Non survive	P value
					N(%)	N(%)	
Total	*N* = 4188		*N* = 124		*N* = 5110	*N* = 477	
**Age groups**							
Under 50	1509(36.0)	< 0.001	37(29.8)	0.001	2432(47.6)	79(16.6)	< 0.001
50 and higher	2679(64.0)		87(70.2)		2678(52.4)	398(83.4)	
**Sex**							
Male	2017(48.2)	< 0.001	52(41.9)	0.082	2570(50.3)	205(43.0)	0.002
Female	2171(51.8)		72(58.1)		2540(49.7)	272(57.0)	
**Co morbidity**							
No	3025(72.2)	< 0.001	77(62.1)	< 0.001	3962(77.5)	315(66.0)	< 0.001
Yes	1163(27.8)		47(37.9)		1148(22.5)	162(34.0)	
**Outcome**							
Survive	3725(88.9)	< 0.001	72(58.1)	< 0.001	–	–	–
Non survive	463(11.1)		52(41.9)		–	–	
**Having contact with a confirmed case of COVID 19 in last 14 days**							
No	4042(98.3)	< 0.001	115(96.6)	0.568	148(3.0)	2(0.4)	0.001
Yes	68(1.7)		4(3.4)		4863(97.0)	470(99.6)	
**Abnormal findings in chest radiography**							
No	3676(87.8)	< 0.001	103(83.1)	0.005	4641(90.8)	413(86.6)	0.003
Yes	512(12.2)		21(16.9)		469(9.2)	64(13.4)	

The distribution of hospitalization, hospitalization in ICU, and the outcome of the disease by the clinical presentation is illustrated in Table 2. As indicated in Table 2 hospitalization have a significant correlation with all symptoms (except diarrhea). This table also shows that hospitalization in ICU has a significant correlation with fever or chills, shortness of breath, aches and pains, runny nose, and chest pain. This table revealed that the outcome of the disease has a significant correlation with all symptoms except cough and sore throat.

**Table 2:** Clinical presentation according to the disease status in Ardabil province, Northwest of Iran,2020

Items	Hospitalization		Hospitalization in ICU		Outcome		
	Yes (%)	P value	Yes (%)	P value	Survive	Non survive	P value
	N = 4188		N = 124		N = 5110	N = 477	
**Fever or chills**							
No	1732(41.4)	< 0.001	46(37.1)	0.040	2432(47.6)	148(31.0)	< 0.001
Yes	2456(58.6)		78(62.9)		2678(52.4)	329(69.0)	
**Cough**							
No	1059(25.3)	< 0.001	41(33.1)	0.137	1406(27.5)	114(23.9)	0.090
Yes	3129(74.7)		83(66.9)		3704(72.5)	363(76.1)	
**Shortness of breath**							
No	1561(37.3)	< 0.001	24(19.4)	< 0.001	2299(45.0)	108(22.6)	< 0.001
Yes	2627(62.7)		100(80.6)		2811(55.0)	369(77.4)	
**Aches and pains**							
No	2844(67.9)	< 0.001	96(77.4)	0.007	3339(65.3)	352(73.8)	< 0.001
Yes	1344(32.1)		28(22.6)		1771(34.7)	125(26.2)	
**Sore throat**							
No	2463(58.8)	< 0.001	69(55.6)	0.712	2938(57.5)	262(54.9)	0.278
Yes	1725(41.2)		55(44.4)		2172(42.5)	215(45.1)	
**Headache**							
No	3460(82.6)	< 0.001	109(87.9)	0.055	4120(80.6)	419(87.8)	< 0.001
Yes	728(17.4)		15(12.1)		990(19.4)	58(12.2)	
**Runny nose**							
No	3964(94.5)	0.006	110(88.7)	0.001	4885(95.6)	429(89.9)	< 0.001
Yes	224(5.3)		14(11.3)		225(4.4)	48(10.1)	
**Diarrhea**							
No	4018(95.9)	0.130	120(96.8)	0.552	4881(95.5)	466(97.7)	0.025
Yes	170(4.1)		4(3.2)		229(4.5)	11(2.3)	
**Chest pain**							
No	3870(92.4)	< 0.001	95(76.6)	< 0.001	4792(93.8)	413(86.6)	< 0.001
Yes	318(7.6)		29(23.4)		318(6.2)	64(13.4)	

In the univariate analysis, all possible effective factors were interred into the model. As can be seen in Table 3, older patients had higher odds of death. Besides, except for having cough and sore throat, all other variables were related to death. This analysis indicated that having contact with a confirmed case of COVID-19 in the last 14 days, aches and pains, headache and diarrhea have a protective effect on death but other variables increase the odds of death.

**Table 3:** Related factors associated with death due to COVID 19 in Ardabil province, Northwest of Iran,2020

	***Death due to COVID 19***					
	***Crude estimation***			***Adjusted estimation***		
**Variables**	**OR**	**95%CI**	**P**	**OR**	**95%CI**	**P**
Age (50 years old or higher)	4.57	3.57–5.86	< 0.001	3.11	2.39–4.06	< 0.001
Sex (being male)	1.34	1.11–1.62	0.002	1.20	0.98–1.47	0.072
Co-morbidity	1.77	1.45–2.17	< 0.001	1.25	1.00–1.56	0.055
Having contact with a confirmed case of COVID 19 in last 14 days	0.14	0.03–0.57	0.006	0.290	0.07–1.22	0.091
Having abnormal findings in chest radiography	1.53	1.16–2.03	0.003	1.00	0.74–1.35	0.996
Fever or chills	2.02	1.65–2.47	< 0.001	1.61	1.29–2.02	< 0.001
Cough	1.21	0.97–1.50	0.090	0.91	0.71–1.17	0.463
Shortness of breath	2.79	2.24–3.49	< 0.001	1.82	1.44–2.31	< 0.001
Aches and pains	0.67	0.54–0.83	< 0.001	0.71	0.57–0.89	0.003
Sore throat	1.11	0.92–1.34	0.278	–	–	–
Headache	0.58	0.43–0.76	< 0.001	0.64	0.47–0.86	0.004
Runny nose	2.43	1.75–3.37	< 0.001	1.54	1.06–2.24	0.022
Diarrhea	0.50	0.27–0.93	0.028	0.66	0.35–1.26	0.206
Chest pain	2.33	1.75–3.11	< 0.001	1.53	1.10–2.13	0.011
Hospitalization	12.28	7.19–20.99	< 0.001	5.66	3.28–9.78	< 0.001
Hospitalization in ICU	8.56	5.91–12.39	< 0.001	5.12	3.40–7.71	< 0.001

However, the result of the multiple logistic regression model indicated that after adjusting for other factors, higher age (OR = 3.11), fever or chills (OR = 1.61), shortness of breath (OR = 1.82), fatigue (OR = 0.71), headache (OR = 0.64), runny nose (OR = 1.54), Skeletal muscle pain (OR = 1.53), hospitalization (OR = 5.66), and hospitalization in ICU (OR = 5.12) were associated with death. In other words, among these related factors, having extra-pulmonary symptoms decreased the odds of death. However other factors are related to increased odds of death due to COVID-19 in all patients.

## Discussion

In this study, we reported the characteristics of all patients with COVID 19 in Ardabil province in the northwest of Iran. The clinical characteristics of these patients showed that age (50-year-old and higher), hospitalization, and hospitalization in ICU were the most important risk factors for death.

The present study also indicated that there is no gender difference in the prevalence of COVID-19. However, of deceased patients most of them were female. Some studies have reported a greater prevalence of COVID-19 in males [16, 18, 19]. A study conducted in Iran showed that males are at higher risk of mortality [20]. But, gender differences seem to have less importance as a prognostic factor for death compared to age [19]. Some studies also have indicated that the susceptibility of older males is higher than older females [21]. Anyway, in this study male gender didn’t have a significant effect on the death of patients in the final adjusted regression logistic model.

Several studies have indicated that comorbidities are one the strongest predictors of death or severe COVID-19 [22–24]. Another study from Iran showed that having comorbidities had a significant effect on mortality [20]. Our findings showed that having any comorbidity was associated with hospitalization, hospitalization in ICU, and outcome of the disease. Also, in univariate analysis comorbidity increased the odds of death. However, this association didn’t remain significant in the final model. Further investigations are needed to assess the role of comorbidities on the mortality of COVID 19 in Iran.

Pulmonary symptoms such as cough and shortness of breath were to be the most common symptom among hospitalized patients. We found that fever or chills, shortness of breath, fatigue, headache, runny nose, diarrhea, and Skeletal muscle pain were factors independently associated with mortality when adjusted for other variables. Among these symptoms, shortness of breath had the strongest effect on death. Some studies reported other frequencies for disease symptoms. For example among Italian and Spanish patients fever was reported as the most common symptom [19, 25]. Probably there are complex interactions between symptoms of COVID-19 and other independent variables like age and gender. Identifying such a complex relationship among independent variables may help to determine the portion of each symptom in death.

Our findings revealed that hospitalizing and hospitalization in ICU increased the odds of death among all patients. In line with our results, a study from Mexico reported that hospitalization (OR = 5.02) and hospitalization in ICU (OR = 1.79) were associated with death [26]. This could happen due to the condition of patients at admission time. Patients with severe and critical conditions are more likely to be hospitalized or hospitalized in ICU, resulting in high in-hospital mortality.

The strength of this study was its large sample that increases the generality of the findings. However, it had some limitations, too. One of these limitations was using self-reported information about some aspects of COVID-19 such as symptoms and history of contact with confirmed COVID-19. Additionally, this study was unable to explain the causal relationship between independent variables and death due to COVID-19. Some unknown factors and possible biases could distort the findings.

## Conclusion

This study revealed that most of the COVID-19 deceased patients were older than 50 years old. Hospitalization had the strongest effect on mortality followed by hospitalization in ICU, and higher age. This study showed that having extra-pulmonary symptoms like skeletal muscle pain, fatigue, and headache can serve as good prognostic factors for mortality. This study showed that having some pulmonary symptoms in contrast with extra-pulmonary symptoms can predict poor prognostic factors of mortality.

## Data Availability

The datasets generated and/or analysed during the current study are not publicly available due Ethics Committee of Ardabil University of Medical Sciences restrictions but are available from the corresponding author on reasonable request.

## Abbreviations

COVID-19: Coronavirus Disease 2019
OR: odds ratio
ICU: Intensive Care Unit
RNA: ribonucleic acid
WHO: World Health Organization
SARS-CoV: Severe acute respiratory syndrome coronavirus 2
